# HEALTH CARE COST DIFFERENCES BY GEOGRAPHIC CONTEXT AND DEMENTIA STATUS FOR MEXICANS AND MEXICAN AMERICANS

**DOI:** 10.1093/geroni/igae098.0047

**Published:** 2024-12-31

**Authors:** Mónika López Anuarbe, Angela Gutierrez

**Affiliations:** Connecticut College, New London, Connecticut, United States; Western University of Health Sciences, Pomona, California, United States

## Abstract

Research documents geographic differences in healthcare costs among Latinés living in the U.S. Yet healthcare costs by geographic context and dementia status for Latiné subpopulations residing in different countries, such as Mexicans in the U.S. and in México, have been overlooked. Utilizing pooled cross-sectional and weighted data of Mexicans ages 50+ from the 2012-2015 Mexican Health and Aging Study (n=15,723) and the 2012-2016 Health and Retirement Study (n=5,136), we investigated: 1) Associations between geographic context and healthcare costs; 2) Whether dementia status moderates the geographic context-healthcare costs relationship. We hypothesized that persons with dementia incurred higher healthcare costs in both countries, and that residents in less populated areas had higher healthcare costs. Preliminary findings from our two-part model (logit; GLM), which accounted for personal and healthcare characteristics, propose that persons with dementia in México had 60.3% higher average healthcare costs than individuals without dementia. Respondents with dementia also had higher odds (b=0.12; p=0.04) of incurring any healthcare costs in less densely populated areas (under 15,000 persons). However, their U.S. counterparts with dementia had lower, but insignificant, odds of incurring healthcare costs, with the dementia-rural interaction suggesting that individuals with dementia in areas with at 250,000-1 million persons were less likely to incur healthcare costs compared to areas with at least 1 million inhabitants (p<0.001), and that their healthcare costs were lower (p=0.05).These results underscore how geographical context varies across countries and how dementia status leaves some individuals more financially vulnerable than others, affecting healthcare access and overall health.

